# Associations between smoking and coronary heart disease: mediating role of RDW

**DOI:** 10.3389/fpubh.2024.1447303

**Published:** 2024-11-15

**Authors:** Mingfeng Ma, Yue Wu, Xingmin He, Miaomiao Zhang, Yanlin Han, Renwei Guo, Shaojie Li

**Affiliations:** ^1^Department of Cultivation Key Laboratory of Metabolic Cardiovascular Diseases Research, Fenyang Hospital Affiliated to Shanxi Medical University, Fenyang, Shanxi, China; ^2^Department of Cardiovascular Medicine, Fenyang Hospital Affiliated to Shanxi Medical University, Fenyang, Shanxi, China; ^3^Fenyang College of Shanxi Medical University, Fenyang, Shanxi, China

**Keywords:** coronary heart disease, smoking, red blood cell distribution width, NHANES, mediation analysis

## Abstract

**Introduction:**

Previous studies have demonstrated that both smoking and coronary heart disease (CHD) are linked to red cell distribution width (RDW). However, the role of the RDW in the association between smoking and CHD remains unclear. This study investigates the role of RDW in the association between smoking and coronary heart disease.

**Methods:**

Data from 13,080 adults aged 40–80 years were analyzed from the National Health and Nutrition Examination Survey (NHANES) conducted between 2006 and 2017. Statistical methods included regression analysis, restricted cubic spline curve (RCS), subgroup analysis, and mediation analysis.

**Results:**

Results showed higher RDW levels in participants with smoking and/or CHD than in those without. Smoking status was positively associated with RDW and CHD even after adjusting for potential confounders. A nonlinear relationship between RDW and CHD was observed (*P* for nonlinear <0.001). Subgroup analysis revealed that sex influenced the relationship between smoking and CHD (*p* = 0.0284). Mediation analysis showed that increased RDW levels mediated the association between smoking status and CHD (PM = 2.1959%, ACME = 0.000694, 95% CI = 0.000262–0.001259, *p* < 0.001).

**Discussion:**

Our research showed that smoking, RDW, and CHD are interrelated, with RDW playing as a mediator, offering a novel perspective for the prevention and management of CHD.

## 1 Introduction

Cardiovascular diseases (CVD) pose a significant global threat to human health, with increasing prevalence and mortality rates annually (1). Among these, coronary heart disease (CHD), encompassing conditions such as angina pectoris, myocardial infarction, ischemic cardiomyopathy, and progressive coronary artery disease, stands as the leading cause of death and a significant contributor to disability within CVD (2–4). Therefore, identifying effective and controllable clinical indicators to predict or prevent the onset of CHD is crucial.

Smoking is consistently recognized as a primary modifiable behavioral risk factor for CHD (5, 6). It increases the risk of coronary atherosclerosis, thereby exacerbating the risk and clinical manifestations of CHD. Studies have found that quitting smoking and reducing cigarette consumption can mitigate the risk of stroke and myocardial infarction, with earlier cessation being more beneficial for survival (7). Despite widespread awareness of its health hazards, smoking prevalence remains high in most countries (8), presenting a significant public health challenge.

Red blood cell distribution width (RDW) serves as a critical blood marker in the pathogenesis of CHD (9, 10). RDW measures the variability and heterogeneity of red blood cells in the circulation and forms an integral part of the standard complete blood count (CBC) (11). Elevated RDW levels are associated with impaired erythropoiesis and heightened red blood cell degradation, leading to compromised blood circulation and oxygen delivery, ultimately contributing to adverse outcomes. Previous studies have shown that RDW can effectively predict the incidence and mortality of various cardiovascular diseases, with higher RDW levels closely associated with the onset and progression of CHD (12–14).

Several studies suggest that smoking is linked to increased RDW levels. Smokers tend to exhibit higher average RDW values compared to nonsmokers, with RDW significantly positively correlated with both the daily quantity of cigarettes smoked and the duration of smoking (15). For instance, one study found evidence that elevated RDW could be attributed to the oxidative stress and inflammation caused by smoking, which disrupts erythropoiesis and promotes red blood cell turnover (16).

However, few studies have investigated whether RDW mediates the association between smoking and CHD. This study addressed this gap in the literature by analyzing data from a public database to explore whether RDW mediates the relationship between smoking and CHD. Specifically, we hypothesized that smoking increases RDW levels, which in turn elevates the risk of CHD. By elucidating the mediating role of RDW, this research aimed to provide new insights into the clinical management of CHD and highlight the importance of RDW as a potential target for intervention in smokers.

## 2 Materials and methods

### 2.1 Study population

This study conducted a cross-sectional analysis using survey data from the National Health and Nutrition Examination Survey (NHANES) collected between 2006 and 2017. Ethical approval for this survey was granted by the Ethics Committee of the Center for Disease Control and Prevention (CDC), and the NHANES study protocol was authorized by the Research Ethics Review Board of the National Center for Health Statistics in accordance with the revised Declaration of Helsinki. Informed consent was obtained from all participants. Detailed information on datasets, documentation, and protocols can be accessed freely on the NHANES website.^1^ Data privacy was maintained through strict adherence to confidentiality protocols to ensure that participant information was de-identified.

**Figure 1:**
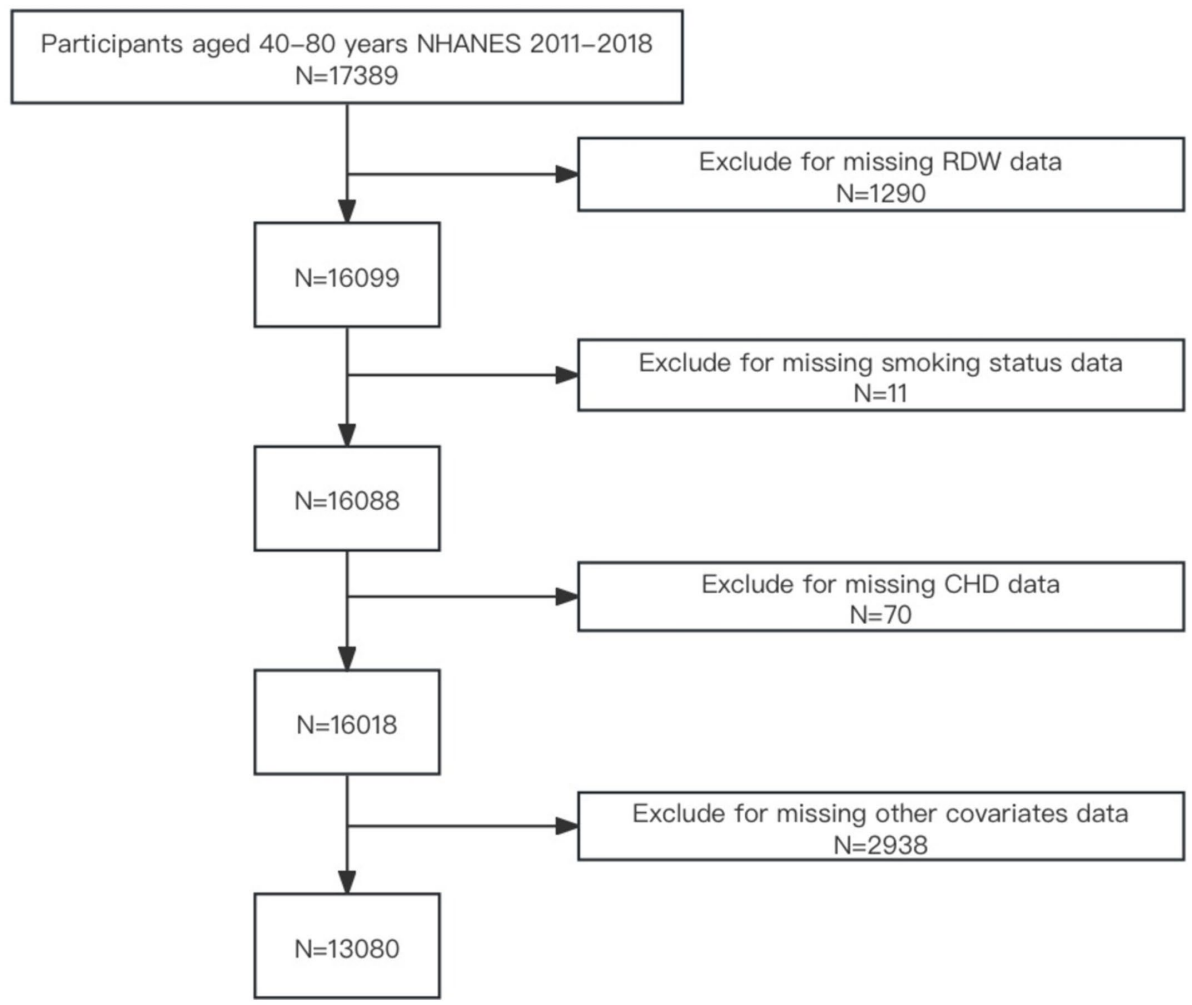
Participant selection flowchart.

Given that age is a significant risk factor for cardiovascular disease, particularly among individuals aged over 40 years. This age group is a primary focus for related research and prevention efforts (17, 18). Due to the complex health statuses often observed in individuals aged over 80 years attributed to the coexistence of multiple diseases (19, 20), this cross-sectional analysis was confined to individuals aged 40–79 years (*n* = 17,389). From this initial cohort, 4,309 individuals were excluded due to missing data on RDW (*n* = 1,290), smoking status (*n* = 11), CHD (*n* = 70), or other covariates such as demographic variables, body mass index (BMI), alcohol use, diabetes, hypertension, hyperlipidemia (*n* = 2,938). Ultimately, the final sample comprised 13,080 participants. A flowchart depicting participant selection is provided in Figure 1.

### 2.2 Exposure: smoking

Trained interviewers conducted smoking-related questions during the NHANES Mobile Examination Center interviews using a computer-assisted personal interview system. Based on their self-reported responses to the question, “Have you smoked at least 100 cigarettes in your lifetime?” participants were categorized into two groups: “nonsmokers” (those who answered “No”) and “smokers.” (those who answered “Yes”).

### 2.3 Outcomes: CHD

The outcomes of this study were derived from self-reported diagnoses in a CVD-related questionnaire. Participants who reported a diagnosis of CHD, MI, or angina were included in the CHD group for our research (21).

### 2.4 Laboratory measures

Blood samples were collected at the NHANES Mobile Examination Center. RDW (%) was measured using the Beckman Coulter MAXM instrument from 2007 to 2012 and the Beckman Coulter DXH 800 instrument from 2013 to 2016, combined with an automatic dilution and mixing device for sample processing.

### 2.5 Covariates

The choice of covariates was based on established risk factors for CHD and their potential to confound the relationship between smoking, RDW, and CHD. Age was included as a continuous variable. Race (non-Hispanic white, Mexican American, non-Hispanic black, other Hispanic, or other race/multiracial), education level (less than high school, high school or GED, or more than high school), marital status (married/living with partner, widowed/divorced/separated, or never married), poverty-income ratio (PIR) (low, middle, high), BMI(normal weight, over-weight, obese), alcohol consumption (current, former, never), diabetes status (yes, no), hypertension status (yes, no), and hyperlipidemia status(yes, no) were considered as categorical variables.

Income levels were categorized into three tiers based on the ratio of family income to poverty (PIR): low (<1.30), middle (1.30–3.49), and high (≥ 3.50) (22). BMI was calculated using weight (kg) and height (kg/m2) and categorized into the following classes: Normal weight (< 25), Overweight (23–28), and Obese (≥ 30) (29). Alcohol status was categorized as never drinkers (lifetime alcohol <12 drinks), former drinkers (lifetime alcohol ≥12 drinks but last year alcohol <12 drinks), or current drinkers (lifetime alcohol ≥12 drinks and last year alcohol ≥12 drinks) (30). Diabetes mellitus (DM) was defined as hemoglobinA1c ≥6.5%, fasting plasma glucose ≥7.0 mmol/L, a self-reported physician diagnosis, or current use of antidiabetic medication or insulin (23). Hypertension was defined as systolic blood pressure (SBP) ≥140 mmHg, diastolic blood pressure (DBP) ≥90 mmHg, a self-reported physician diagnosis, or current use of antihypertensive medication (24). Hyperlipidemia was defined as triglycerides (TG) ≥150 mg/dL, total cholesterol (TC) ≥200 mg/dL, high-density lipoprotein cholesterol (HDL-C) <40 mg/dL, low-density lipoprotein cholesterol (LDL-C) ≥130 mg/dL, a self-reported physician diagnosis, or current use of lipid-lowering medication (25, 26).

### 2.6 Statistical analysis

Continuous variables following a normal distribution were presented as mean ± standard deviation (SD), while non-normally distributed continuous variables were presented as median and interquartile range (IQR). Categorical variables were reported as proportions and percentages. Differences between groups were assessed using one-way ANOVA for normally distributed data, the Kruskal-Wallis test for non-normally distributed data, and the chi-square test for categorical variables.

Multivariate logistic regression was used to evaluate the relationships among smoking, RDW, and CHD, while multivariate linear regression analysis assessed the relationship between smoking status and RDW. Two models were developed for the analysis: Model 1 was unadjusted for confounders, representing a univariate analysis. Model 2 was adjusted for age, sex, race, education, PIR, BMI, diabetes, hypertension, and hyperlipidemia.

RCS based on logistic regression models was used to visualize the relationship between RDW and CHD according to Model 2, with knots placed at the 10th, 50th, and 90th percentiles of RDW. Threshold levels for each section were determined using likelihood ratio tests and bootstrap resampling. A segmented regression model was further applied to examine the relationship between RDW and CHD.

Stratified subgroup and interaction analyses were conducted using multivariate logistic regression with full adjustment for categorical variables.

Finally, mediation analysis was performed to examine whether RDW mediated the relationship between the exposure variable (smoking) and outcome (CHD). The analysis used 1,000 bootstrap samples to display the size of the indirect path effect, the proportion of the mediation effect, and the *p*-value of the mediation effect in the results.

All statistical analyses were performed using statistical software (R version 4.3.3). Descriptive statistics were obtained using the dplyr package, RCS analysis using the plotRCS package, segmented regression analyses using the segmented package, forest plots using the forestplot package, mediation analysis using the mediation package, bootstrap resampling methods using the boot package, and graph plots using the ggplot2 package. A *p*-value less than 0.05 was considered statistically significant in all analyses.

## 3 Results

### 3.1 Participant characteristics

A total of 13,080 participants were enrolled in this study, of whom 49.23% were smokers, and 8.99% were diagnosed with CHD. Participants (*n* = 13,080) were divided into four groups: “smoke with CHD” (*n* = 768), “smoke without CHD” (*n* = 5,672), “CHD without smoke” (*n* = 408), and “without smoke and CHD” (*n* = 6,232). None of the continuous variables in our data were normally distributed. As shown in Table 1, the median age of participants was 57 years, and 49.2% were male. The racial distribution was as follows: 14.8% Mexican American, 44.1% non-Hispanic white, 21.3% non-Hispanic black, and 19.8% other. Educational levels were 26.1% less than high school, 22.6% high school or GED, and 51.3% higher than high school. Marital status distribution was: 63.9% married or living with a partner, 27.6% widowed, divorced, or separated, and 8.5% never married. BMI categories were 23.8% normal weight, 34.4% overweight, and 41.8% obese. PIR distribution was 29.9% low, 36.0% medium, and 34.1% high. Drinking status was 70.9% current, 14.9% former, and 14.3% never. The prevalence rates of diabetes, hypertension, and hyperlipidemia were 23.9, 53.5, and 78.6%, respectively.

**Table 1:** Characteristics of study participants (*n* = 13,080).

Characteristic	Total participants	Smoke with CHD	Smoke without CHD	CHD without smoke	Without smoke and CHD	*p*-value
	(*n* = 13,080)	(*n* = 768)	(*n* = 5,672)	(*n* = 408)	(*n* = 6,232)	
Age, y, median (IQR)	57.00 [48.00, 66.00]	65.00 [58.00, 72.00]	57.00 [49.00, 66.00]	65.00 [58.00, 72.00]	55.00 [47.00, 64.00]	<0.001
Gender, n (%)						<0.001
Male	6,431 (49.2)	522 (68.0)	3,254 (57.4)	217 (53.2)	2,438 (39.1)	
Female	6,649 (50.8)	246 (32.0)	2,418 (42.6)	191 (46.8)	3,794 (60.9)	
Race, n (%)						<0.001
Mexican American	1936 (14.8)	70 (9.1)	758 (13.4)	63 (15.4)	1,045 (16.8)	
Non-Hispanic White	5,763 (44.1)	442 (57.6)	2,740 (48.3)	170 (41.7)	2,411 (38.7)	
Non-Hispanic Black	2,792 (21.3)	151 (19.7)	1,237 (21.8)	80 (19.6)	1,324 (21.2)	
Other	2,589 (19.8)	105 (13.7)	937 (16.5)	95 (23.3)	1,452 (23.3)	
Education, n (%)						<0.001
Less than high school	3,418 (26.1)	272 (35.4)	1,600 (28.2)	122 (29.9)	1,424 (22.8)	
High school or GED	2,953 (22.6)	200 (26.0)	1,412 (24.9)	85 (20.8)	1,256 (20.2)	
Above high school	6,709 (51.3)	296 (38.5)	2,660 (46.9)	201 (49.3)	3,552 (57.0)	
Marital, n (%)						<0.001
Married/Living with partner	8,359 (63.9)	451 (58.7)	3,454 (60.9)	253 (62.0)	4,201 (67.4)	
Widowed/Divorced/Separated	3,606 (27.6)	264 (34.4)	1707 (30.1)	134 (32.8)	1,501 (24.1)	
Never married	1,115 (8.5)	53 (6.9)	511 (9.0)	21 (5.1)	530 (8.5)	
BMI, n (%)						<0.001
Normal weight	3,114 (23.8)	144 (18.8)	1,466 (25.8)	67 (16.4)	1,437 (23.1)	
Over weight	4,503 (34.4)	233 (30.3)	2017 (35.6)	121 (29.7)	2,132 (34.2)	
Obese	5,463 (41.8)	391 (50.9)	2,189 (38.6)	220 (53.9)	2,663 (42.7)	
PIR, n (%)						<0.001
Low	3,913 (29.9)	336 (43.8)	1869 (33.0)	161 (39.5)	1,547 (24.8)	
Middle	4,705 (36.0)	278 (36.2)	2070 (36.5)	129 (31.6)	2,228 (35.8)	
High	4,462 (34.1)	154 (20.1)	1733 (30.6)	118 (28.9)	2,457 (39.4)	
Alcohol, n (%)						<0.001
Current	9,269 (70.9)	627 (81.6)	4,808 (84.8)	225 (55.1)	3,609 (57.9)	
Former	1944 (14.9)	95 (12.4)	619 (10.9)	89 (21.8)	1,141 (18.3)	
Never	1867 (14.3)	46 (6.0)	245 (4.3)	94 (23.0)	1,482 (23.8)	
DM, n (%)						<0.001
No	9,952 (76.1)	432 (56.2)	4,385 (77.3)	213 (52.2)	4,922 (79.0)	
Yes	3,128 (23.9)	336 (43.8)	1,287 (22.7)	195 (47.8)	1,310 (21.0)	
Hypertension, n (%)						<0.001
No	6,093 (46.6)	169 (22.0)	2,680 (47.2)	77 (18.9)	3,167 (50.8)	
Yes	6,987 (53.4)	599 (78.0)	2,992 (52.8)	331 (81.1)	3,065 (49.2)	
Hyperlipidemia, n (%)						<0.001
No	2,803 (21.4)	90 (11.7)	1,188 (20.9)	56 (13.7)	1,469 (23.6)	
Yes	10,277 (78.6)	678 (88.3)	4,484 (79.1)	352 (86.3)	4,763 (76.4)	
RDW, %, median (IQR)	13.10 [12.50, 13.80]	13.50 [12.88, 14.40]	13.10 [12.50, 13.90]	13.30 [12.80, 14.10]	13.10 [12.50, 13.80]	<0.001

BMI, body mass index; PIR, the ratio of family income to poverty; DM, Diabetes mellitus; RDW, Red cell distribution width.

Compared to the “without smoke and CHD” group, the “smoke with CHD” group tended to be slightly older, more likely to be female, more likely to be non-Hispanic white, have a lower PIR, and more likely to have DM, hypertension, hyperlipidemia, as well as higher RDW levels. Additionally, the median RDW level was 13.1%. There were significant differences in RDW levels among the groups: “smoke with CHD,” “smoke without CHD,” “CHD without smoke,” and “without smoke and CHD” (RDW: 13.50 [12.88, 14.40] vs. 13.10 [12.50, 13.90] vs. 13.30 [12.80, 14.10] vs. 13.10 [12.50, 13.80], *p* < 0.001).

### 3.2 Association analysis

The correlations of smoking with RDW and CHD in both crude and adjusted models are summarized in Table 2. Following adjustment for potential confounders (age, sex, race, education, PIR, BMI, alcohol consumption, DM, hypertension, and hyperlipidemia), smoking was found to be significantly associated with CHD (OR = 1.56, 95% confidence interval[CI] = 1.36–1.80, *p* < 0.001). Moreover, smoking was associated with RDW (OR = 0.08, 95%CI = 0.03–0.13, *p* = 0.001).

**Table 2:** Associations of smoking with RDW and CHD (*n* = 13,080).

	OR (95%CI)	*p*-value
Associations of smoke with CHD		
Unadjusted Model	2.07 [1.83, 2.35]	<0.001
Adjusted Model	1.56 [1.36, 1.80]	<0.001
Associations of smoke with RDW		
Unadjusted Model	0.07 [0.02, 0.12]	0.004
Adjusted Model	0.08 [0.03, 0.13]	0.001

Unadjusted Model: no adjustment for covariates; adjusted model: adjusted for age, sex, race, education, PIR, BMI, alcohol, DM, hypertension, and hyperlipidemia.

### 3.3 Restricted cubic spline curve fitting and segmented regression analyses

**Figure 2:**
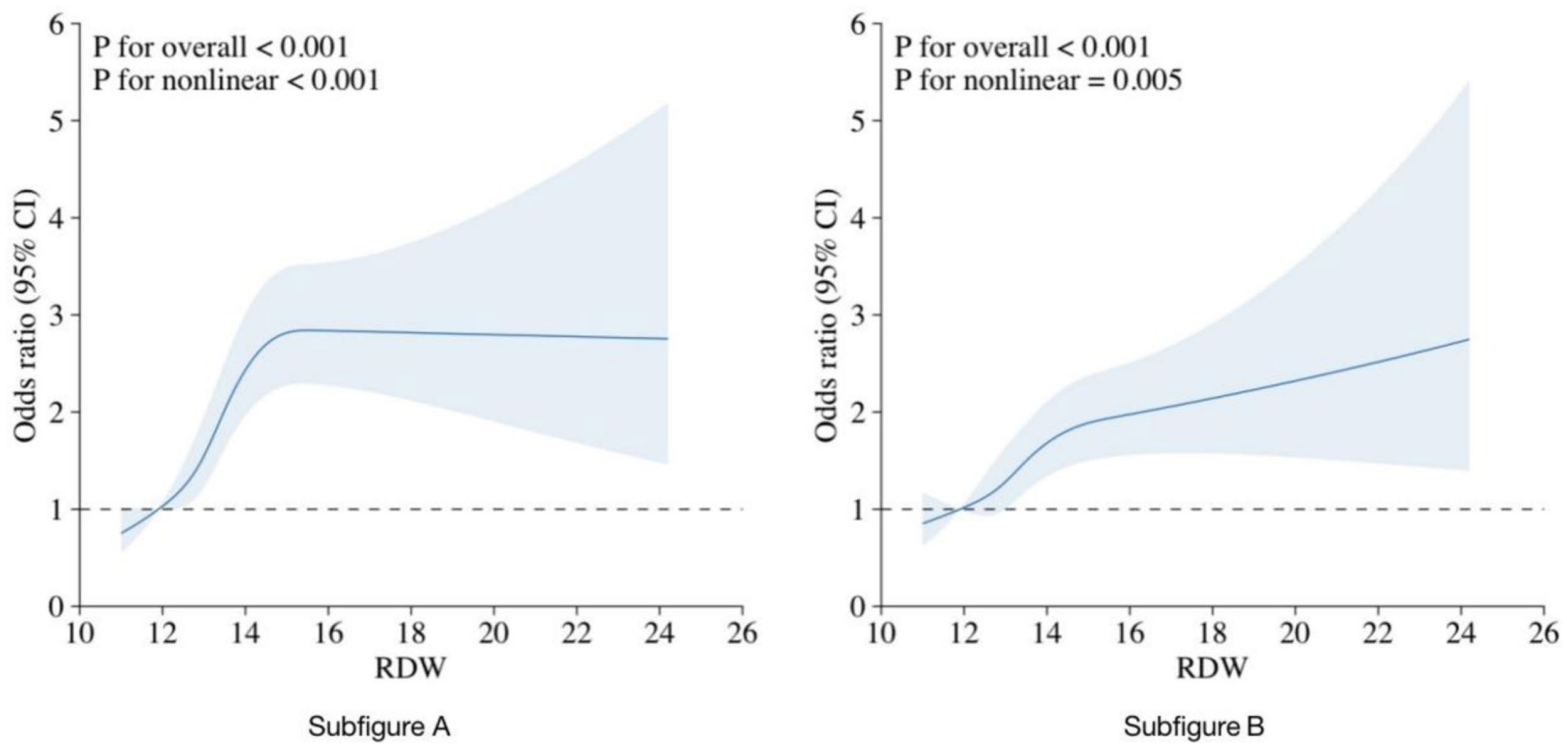
RCS curve fit between RDW and CHD. Notes: Solid lines represent smooth curve fits between the variables. The bands represent the 95%CI from the fit. (A) No adjustment for covariates. (B) Adjusted for age, sex, race, education, PIR, BMI, alcohol consumption, DM, hypertension, and hyperlipidemia.

Figure 2 depicts the relationship between RDW and CHD using restricted cubic spline curves. Subfigures A and B represent the crude and adjusted models, respectively. Both models showed a nonlinear relationship between RDW and CHD (*P* for nonlinear <0.001 for the crude model and *p* = 0.005 for the adjusted model).

A threshold effect analysis on the adjusted model identified an RDW cutoff point of 14.5% as the best fit. The risk of CHD rapidly increased with increasing RDW up to this cutoff point and then rose more steadily. The relationship between RDW and CHD remained significant both before and after the cutoff point (*p* < 0.001). As shown in Table 3, when RDW was <14.5%, a 1 SD increase in RDW was associated with an OR of 1.29 (95%CI = 1.17–1.43) for CHD, while there was no significant difference between RDW and CHD when RDW was ≥14.5% (OR = 1.03, 95% CI = 0.91–1.18, *p* = 0.711).

**Table 3:** Threshold effect analyzes of association between RDW and CHD using segmented regression models.

	Adjusted OR (95%CI)^a^	*p*-value
Logistic regression models	1.13 [1.08, 1.18]	<0.001
Segmented regression models		
RDW <14.5%	1.29 [1.16, 1.43]	<0.001
RDW ≥14.5%	1.03 [0.91, 1.18]	0.711
Likelihood ratio test		<0.001

RDW, Red cell distribution width.

a Adjusted for age, gender, race, education, PIR, BMI, alcohol, DM, hypertension and hyperlipidemia.

### 3.4 Subgroup analysis

**Figure 3:**
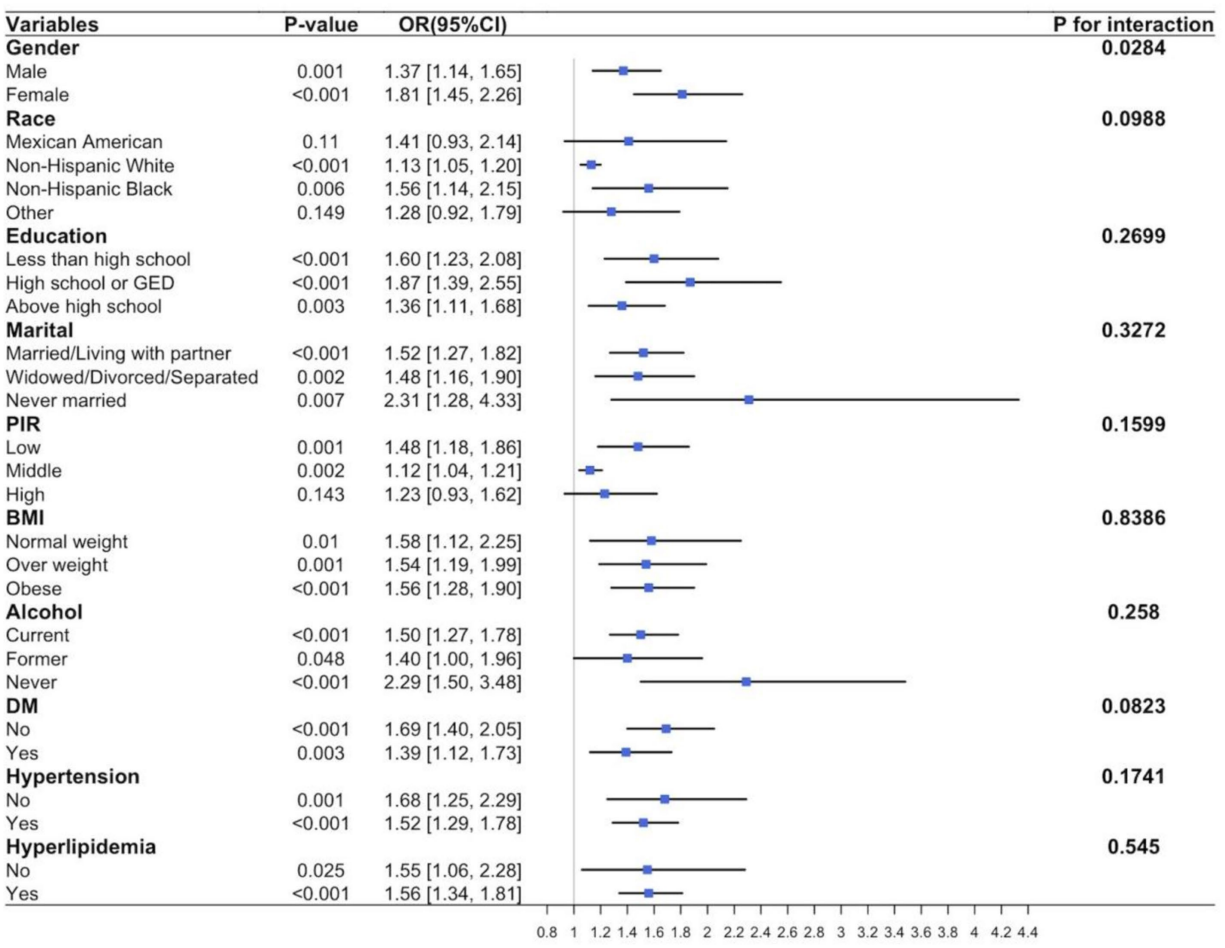
Subgroup analysis for the association between smoking and CHD.

Further subgroup analysis was conducted using the adjusted model to study potential variations in the relationship between smoking and CHD among different populations. The results, shown in Figure 3, indicate a significant positive correlation between smoking and CHD incidence in most subgroups, including sex, race, education, marital status, PIR, BMI, alcohol consumption, diabetes, hypertension, and hyperlipidemia (*p* < 0.001). Exceptions were observed in Mexican Americans (*p* = 0.11), other races (*p* = 0.149), and high-PIR populations (*p* = 0.143). No significant evidence was found regarding the effects of the interactions between smoking and important covariates on the risk of CHD (all *p* ≥ 0.05). Additionally, a significant interaction between sex subgroups in the relationship between smoking and CHD was observed (*p* = 0.0284).

### 3.5 The mediating role of RDW

**Figure 4:**
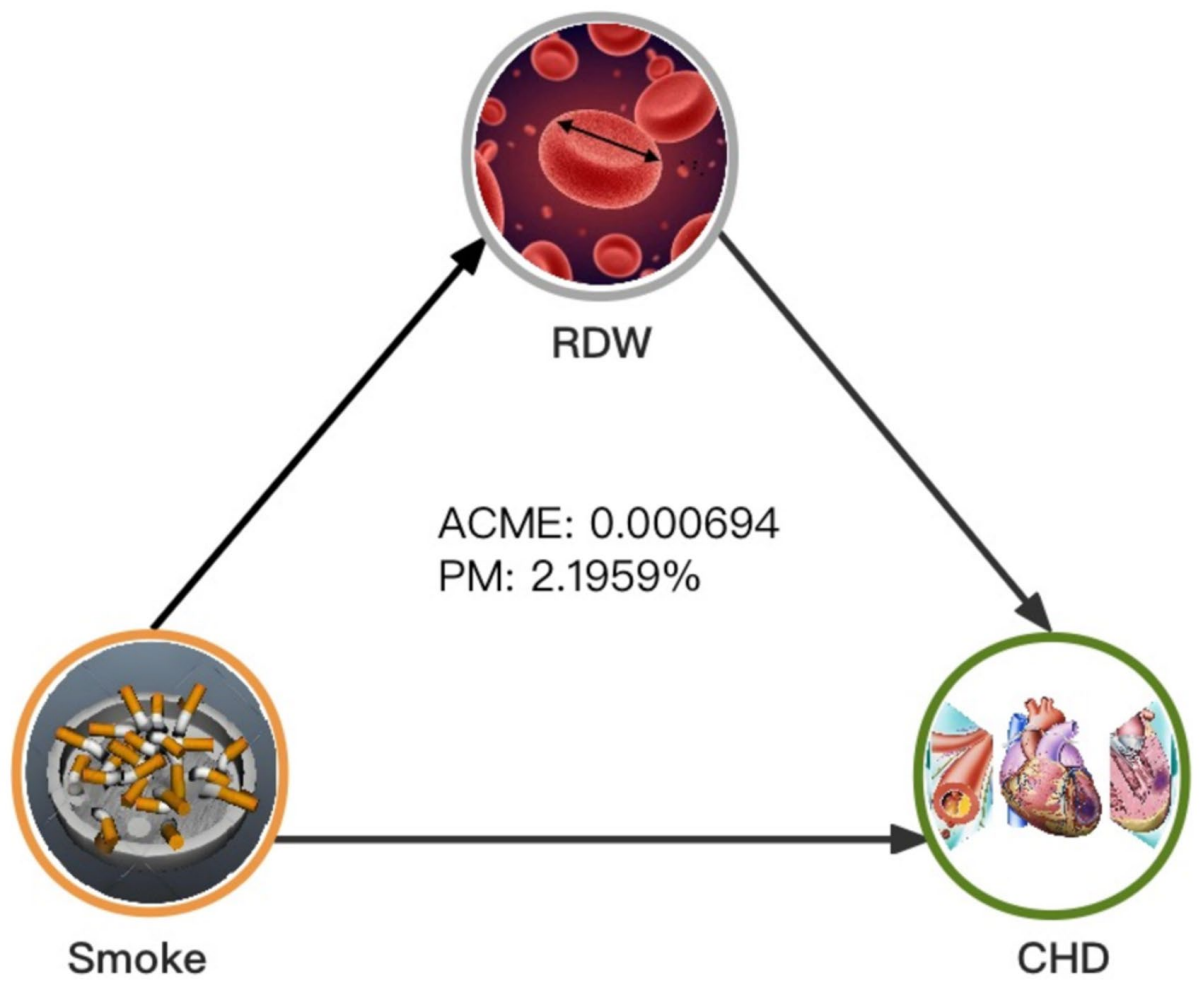
Mediated analysis model path diagram. Notes: Smoking, CHD and RDW are defined as the independent, dependent, and mediating variables, respectively. ACME indicates average causal mediation effects (indirect effect), and PM indicates proportion-mediated effects (e.g., proportion of the total effect due to the mediator). The ACME and average direct effect coefficients are displayed along with PM.

As shown in Table 4 and Figure 4, RDW significantly mediated the association between smoking and CHD (proportion mediated [PM] = 2.1959%, average causal mediation effect [ACME] = 0.000694, 95%CI = 0.000262–0.001259, *p* < 0.001). When participants were divided by sex, RDW significantly mediated the relationship between smoking and CHD in males after adjusting for all covariates (PM = 3.4235%, ACME = 0.000950, 95%CI = 0.000193–0.001916, *p* = 0.012). However, the mediating effect of RDW was not significant in females(PM = 0.7556%, ACME = 0.000256, 95%CI = −0.000115–0.000707, *p* = 0.178). Interestingly, when females were further divided based on their responses to the question, “At what age did you have your last menstrual period?,” RDW significantly mediated the relationship between smoking and CHD in postmenopausal women (PM = 1.8241%, ACME = 0.000732, 95%CI = 0.000159–0.001616, *p* = 0.014), but not in premenopausal women (PM = −0.2854%, ACME = −0.000041, 95%CI = −0.000607–0.000495, *p* = 0.898). The results were smoothed using generalized additive and linear models.

**Table 4:** RDW mediating the association between smoking and CHD (*n* = 13,080).

	ACEM				ADE				Total effect				Proportion mediated			
	Estimate	95%CI lower	95%CI upper	*p*-value	Estimate	95%CI lower	95%CI upper	*p*-value	Estimate	95%CI lower	95%CI upper	*p*-value	Estimate	95%CI lower	95%CI upper	*p*-value
Unadjusted Model																
Total	0.000887	0.000268	0.001554	0.002	0.057112	0.046768	0.067000	<0.001	0.058198	0.047809	0.068000	<0.001	0.015297	0.004502	0.026900	0.002
Male	0.004152	0.002460	0.006000	<0.001	0.051931	0.036340	0.068100	<0.001	0.056083	0.040490	0.072400	<0.001	0.074039	0.043470	0.120600	<0.001
Female	0.000364	−0.000169	0.000978	0.206	0.043923	0.031136	0.056600	<0.001	0.044288	0.031460	0.056800	<0.001	0.008229	−0.004143	0.023130	0.206
menopause	0.001727	0.000759	0.003058	<0.001	0.047332	0.031102	0.064900	<0.001	0.049059	0.032831	0.066500	<0.001	0.035201	0.014657	0.066500	<0.001
premenopause	−0.000326	−0.001063	0.000344	0.338	0.016507	0.001028	0.032550	0.032	0.016182	0.000763	0.031846	0.038	−0.020129	−0.117547	0.039600	0.368
Adjusted Model																
Total	0.000694	0.000262	0.001259	<0.001	0.030923	0.020079	0.041300	<0.001	0.031618	0.020889	0.041900	<0.001	0.021959	0.008017	0.045730	<0.001
Male	0.000950	0.000193	0.001916	0.012	0.026813	0.009732	0.042140	<0.001	0.027764	0.010765	0.043300	<0.001	0.034235	0.005980	0.112770	0.012
Female	0.000256	−0.000115	0.000707	0.178	0.033614	0.020201	0.046600	<0.001	0.033870	0.020615	0.046800	<0.001	0.007556	−0.003365	0.022140	0.178
menopause	0.000732	0.000159	0.001616	0.014	0.039392	0.023158	0.057100	<0.001	0.040124	0.023916	0.057900	<0.001	0.018241	0.004282	0.048720	0.014
premenopause	−0.000041	−0.000607	0.000495	0.898	0.014402	−0.000666	0.030876	0.064	0.014361	−0.000718	0.030809	0.064	−0.002854	−0.078400	0.074700	0.906

BMI, body mass index; PIR, the ratio of family income to poverty; DM, Diabetes mellitus; RDW, Red cell distribution width. RDW were transformed to natural logarithms in the analysis. Generalized additive model was used to smoothen the mediator effect on outcome. Adjusted; analyses adjusted for age, sex, race, education, PIR, BMI, alcohol, DM, hypertension, and hyperlipidemia; sex was adjusted in analyses of the total population but not in subgroups of male or female and menopause or premenopause.

## 4 Discussion

### 4.1 Overview

This study aimed to investigate the association between smoking and CHD in middle-aged and older adult people aged 40–80 in the United States using data from NHANES (2006–2017) and to determine whether RDW partly mediates this association.

### 4.2 Association and mechanisms

Consistent with previous studies (27), our study reaffirmed the positive correlation between smoking and CHD. Even after adjusting for potential confounders, smoking remained a significant risk factor for CHD, emphasizing the urgent need to control smoking to alleviate the burden of cardiovascular diseases.

We also found a correlation between smoking and higher RDW levels, with smokers having higher RDW levels than nonsmokers. We speculated on possible mechanisms underlying the elevation of RDW in smokers. First, smoking has been linked to elevated levels of hs-CRP and other inflammatory markers such as interleukin-6, soluble tumor necrosis factor-alpha, VCAM-1, ICAM-1, and E-selectin (28). Higher RDW values may reflect underlying inflammatory states (12), and significant associations have been observed between CRP levels and RDW (31). Therefore, the increase in RDW may result from ineffective red blood cell production due to chronic inflammation in smokers, leading to the entry of immature red blood cells into circulation and elevated RDW levels. Oxidative stress may be another pathological mechanism linking RDW and smoking. Previous studies have shown an association between smoking and high oxidative stress (32), which can cause red blood cell hemolysis (33). In cases of hemolysis, the reticulocyte count may increase to compensate for the loss of red blood cells, leading to an increase in RDW (34).

Our study revealed a nonlinear relationship between RDW and CHD, with CHD risk increasing significantly up to an RDW cutoff value of 14.5%. Beyond this cutoff value, additional increases in RDW had no substantial effects on CHD risk. The correlation between RDW and inflammatory markers suggests that the association between RDW and CHD may be related to inflammatory reactions. Lappé et al. (35) also reported an association between RDW and CRP levels in patients with coronary artery disease. The study by Ainiwaer et al. (36), which analyzed data from the National Health and Nutrition Examination Survey (1999–2020), further supports these findings. Their study found a significant association between RDW and RDW-to-platelet count ratio with cardiovascular disease (CVD) among US adults, which aligns with our results. They suggest that RDW and related hematological parameters may play a crucial role in cardiovascular health, particularly in populations with a high prevalence of smoking. The present study strengthens the argument that RDW may serve as an important marker for CVD risk and further highlights its potential clinical relevance. These findings indicate that RDW may be a valuable biomarker for identifying individuals at high risk of CHD. These findings are a reminder of the importance of early assessment of RDW levels.

Finally, mediation analysis indicated that RDW partially mediated the relationship between smoking and CHD in middle-aged and older adult individuals, accounting for approximately 2.2% of the total effect. Although the proportion of mediation seems small, it underscores the importance of RDW as a biological pathway through which smoking influences CHD risk. Our subgroup analysis showed a significant effect of sex on the relationship between smoking and CHD. Stratified mediation analysis revealed that the mediating effect of RDW was significant in males but not in females. Further categorization of females based on menopausal status showed a significant mediating effect of RDW in postmenopausal females. This difference suggests that hormonal changes associated with menopause may affect the relationship between smoking, RDW, and CHD. Previous studies have shown that estrogen levels influence biological processes related to atherosclerosis (37). In an atherosclerosis mouse model, estrogen can reduce atherosclerotic plaque burden (38), decrease the upregulation of endothelial cell adhesion molecules induced by cytokines, and reduce interleukin-6 expression, thereby inhibiting inflammation and reducing CHD incidence. Estrogen can also regulate cholesterol reverse transport, lower LDL-C and HDL-C levels, and prevent excessive lipid uptake by macrophages (39). Estrogen also increases endothelial nitric oxide production (40, 41), dilating blood vessels and improving endothelial cell function (42). Additionally, Meng et al. (43) reported that estrogen can reduce vascular endothelial cell pyroptosis through estrogen receptor alpha-mediated activation of autophagy, thus ameliorating atherosclerosis. However, after menopause, ovarian function declines, leading to decreased estrogen levels. When estrogen levels decrease, the efficiency of inflammation inhibition is lower, lipid status is worse than that in premenopausal women, and vascular dilation efficiency is lower. This may exacerbate the impact of smoking on hematological parameters and enhance the mediating role of RDW in this subgroup.

### 4.3 Strengths and limitations of the study

This study is the first to identify the relationship between smoking and CHD mediated by RDW in middle-aged and older adult populations, with a large sample size and diverse ethnic population, enhancing the generalizability of the results. Multiple statistical techniques were used, including regression, restricted cubic spline curve fitting, and mediation analyses, to rigorously examine the relationships among smoking, RDW, and CHD. However, this study has several limitations. First, its cross-sectional design prevents the establishment of causality between smoking, RDW, and CHD, longitudinal studies are needed to confirm the mediating role of RDW and explore underlying mechanisms. Necessitating further longitudinal studies. Second, self-reported methods were used to assess smoking status and CHD, potentially introducing recall and reporting biases. Future studies could incorporate biomarkers (such as nicotine or metabolites in the blood) to validate self-reported data and beter ensure their accuracy. Finally, the RDW was measured at a single time point, failing to capture dynamic changes. Future research should incorporate longitudinal RDW measurements to assess its dynamic relationship with smoking and CHD risk. The findings also need to be confirmed by additional studies on CHD patients with larger a sample size.

### 4.4 Future research and clinical implications

Future research should focus on longitudinal studies to confirm the mediating role of RDW and elucidate the biological mechanisms underlying the relationship between RDW, smoking, and CHD. Exploring the potential clinical applications of RDW as a biomarker for CHD risk is crucial. Public health interventions aimed at reducing smoking and CHD risk should consider incorporating RDW measurements to identify individuals at higher risk. Tailored interventions based on RDW levels could improve preventive strategies and outcomes for populations at risk of CHD.

## 5 Conclusion

Our study highlights an association between RDW, smoking status, and CHD. These findings underscore the importance of RDW as a biomarker linking smoking to an elevated risk of CHD, with a particular emphasis on considering sex hormones in studies involving RDW, smoking, and CHD. Further longitudinal studies are imperative to elucidate whether elevated RDW contributes to the development of optimal intervention strategies tailored to different populations.

## Data Availability

The datasets presented in this study can be found in online repositories. The names of the repository/repositories and accession number(s) can be found in the article/supplementary material.

## Footnotes

^1^http://www.cdc.gov/nchs/nhanes.htm
